# Alarmingly High Segregation Frequencies of Quinolone Resistance Alleles within Human and Animal Microbiomes Are Not Explained by Direct Clinical Antibiotic Exposure

**DOI:** 10.1093/gbe/evv102

**Published:** 2015-05-26

**Authors:** Wesley Field, Ruth Hershberg

**Affiliations:** Rachel & Menachem Mendelovitch Evolutionary Processes of Mutation & Natural Selection Research Laboratory, Department of Genetics and Developmental Biology, the Ruth and Bruce Rappaport Faculty of Medicine, Technion-Israel Institute of Technology, Haifa, Israel

**Keywords:** antibiotic resistance, microbiome, metagenomics, allele frequencies

## Abstract

Antibiotic resistance poses a major threat to human health. It is therefore important to characterize the frequency of resistance within natural bacterial environments. Many studies have focused on characterizing the frequencies with which horizontally acquired resistance genes segregate within natural bacterial populations. Yet, very little is currently understood regarding the frequency of segregation of resistance alleles occurring within the housekeeping targets of antibiotics. We surveyed a large number of metagenomic datasets extracted from a large variety of host-associated and non host-associated environments for such alleles conferring resistance to three groups of broad spectrum antibiotics: streptomycin, rifamycins, and quinolones. We find notable segregation frequencies of resistance alleles occurring within the target genes of each of the three antibiotics, with quinolone resistance alleles being the most frequent and rifamycin resistance alleles being the least frequent. Resistance allele frequencies varied greatly between different phyla and as a function of environment. The frequency of quinolone resistance alleles was especially high within host-associated environments, where it averaged an alarming ∼40%. Within host-associated environments, resistance to quinolones was most often conferred by a specific resistance allele. High frequencies of quinolone resistance alleles were also found within hosts that were not directly treated with antibiotics. Therefore, the high segregation frequency of quinolone resistance alleles occurring within the housekeeping targets of antibiotics in host-associated environments does not seem to be the sole result of clinical antibiotic usage.

## Background

The accumulation and spread of antibiotic resistance is one of the most pressing medical concerns currently facing humanity. Antibiotic resistance can be acquired by a bacterium in two ways:

- Antibiotic resistance can be obtained by acquisition, via horizontal gene transfer, of antibiotic resistance genes that deactivate the antibiotic, prevent its entrance into the cell, alter its target, or remove it from the cell (Schmieder and Edwards 2012). Quantifying the frequencies with which such antibiotic resistance genes are found in various environments is an area of intensive study (e.g., Gilliver et al. 1999; Osterblad et al. 2001; Martinez 2008; Allen et al. 2010; de Vries et al. 2011; Boulund et al. 2012; Hu et al. 2013; Martinez et al. 2015).
- Antibiotic resistance can also be obtained through the acquisition of resistance conferring alleles within an antibiotic’s target gene (Andersson and Hughes 2010). For convenience sake, we will from now on refer to such resistance alleles occurring within the targets of antibiotics as target resistance alleles (TRAs). Such resistance alleles can be acquired either via de novo mutation or via recombination. TRAs may hinder binding of the antibiotic to its target or affect the regulation of the gene, causing the antibiotic to be less effective. Antibiotic usage will impose strong selection in favor of antibiotic resistant bacteria. Therefore, it is expected that the frequency TRAs will be strongly affected by antibiotic exposure. At the same time, because the genes targeted by antibiotics tend to be important housekeeping genes, crucial for bacterial survival, resistance alleles within these genes can have strong phenotypic and fitness effects, independent of antibiotic exposure (Wrande et al. 2008; Paulander et al. 2009; Andersson and Hughes 2010; Kunz et al. 2012; Katz and Hershberg 2013; Miskinyte and Gordo 2013; Qi et al. 2014). Such strong fitness effects could greatly influence the frequencies with which TRAs segregate, in a manner that is independent of antibiotic exposure. The fitness effects of TRAs may vary depending on the specific resistance allele, the identity of the bacterium in which these alleles reside and on environment. It is thought that in most instances, resistance alleles within important housekeeping genes will tend to have harmful fitness effects (Andersson and Hughes 2010). However, several studies have shown that some TRAs can also be adaptive under certain conditions (Wrande et al. 2008; Paulander et al. 2009; Andersson and Hughes 2010; Kunz et al. 2012; Katz and Hershberg 2013; Miskinyte and Gordo 2013; Qi et al. 2014). Additionally, when certain TRAs confer a negative fitness effect on the bacteria carrying them, compensatory mutations can alleviate these effects (reviewed in Andersson and Hughes [2010]). It is likely that the ease with which harmful fitness effects of TRAs can be alleviated will also vary between different alleles, in different bacteria, and within different environments. Differences in the antibiotic-independent fitness effects of TRAs and in the ease with which such fitness effects can be compensated for could influence the frequencies with which various TRAs segregate among varying environments and within different bacterial phyla, independently of levels of antibiotic exposure.

Currently very little is known about the frequencies with which resistance alleles occurring within the target genes of antibiotics segregate within natural bacterial populations. Characterizing these frequencies is the aim of this study. We focused on quantifying the frequency of TRAs conferring resistance to three broad-spectrum antibiotic classes: aminoglycosides (represented by streptomycin), rifamycins, and quinolones. These three antibiotic classes all target important, housekeeping genes that are globally conserved across bacteria. Streptomycin is an aminoglycoside that targets the bacterial 30S ribosomal subunit and disrupts the initiation and elongation steps of protein synthesis, eventually leading to cell death. Certain alleles within the ribosomal gene *rpsL* confer resistance to this antibiotic (table 1) (Ballif et al. 2012). Rifamycins target the beta subunit of RNA polymerase (encoded by the *rpoB* gene) and inhibit bacterial RNA synthesis (Qiu et al. 2012). Certain alleles within *rpoB* confer resistance to rifamycins (table 1) (Bahrmand et al. 2009). Quinolones target the ligation domain of DNA gyrase subunit A (encoded by the *gyrA* gene) and Topoisomerase IV subunit A (encoded by the *parC* gene), preventing the resealing of DNA after double-stranded breaks leading to an accumulation of DNA breaks and eventual cell death (Shen et al. 1989). While quinolones target DNA gyrase more exclusively in Gram-negative bacteria, it is still synergistically targeted in Gram-positive bacteria, along with its paralog, topoisomerase IV (Pan and Fisher 1997). The alpha subunits of DNA gyrase and topoisomerase IV, GyrA and ParC respectively, are highly similar in sequence within the first 300 amino acids, which encompasses their resistance-determining region (RDR). Certain changes to the binding domain of these proteins decrease the binding affinity of these antibiotics and thus confer antibiotic resistance (table 1) (Yoshida et al. 1990). TRAs vary in their impact on binding affinity depending on the physical properties of the substituted amino acid and on location within the RDR. Bacteria harboring multiple resistance alleles within the RDR are typically resistant to higher antibiotic concentrations (Bagel et al. 1999).

**Table 1:** Summary of Antibiotic Resistance Alleles Analyzed in This Study

Antibiotic	Gene	Resistance Allele	Known to Confer Resistance in (Citations)
Streptomycin	*rpsL*	43N	Proteobacteria (Silva et al. 2011; Angst and Hall 2013; Miskinyte and Gordo 2013), Actinobacteria (Okamoto-Hosoya et al. 2003)
		43R	Proteobacteria (Silva et al. 2011; Wang et al. 2011; Miskinyte and Gordo 2013), Deinococcus-Thermus (Carr et al. 2006), actinobacteria (Okamoto-Hosoya et al. 2003)
		88R	Actinobacteria (Okamoto-Hosoya et al. 2003; Wang et al. 2011), Proteobacteria (Angst and Hall 2013)
		88Q	Actinobacteria (Ballif et al. 2012), Proteobacteriajanne (Toivonen et al. 1999)
		91S	Proteobacteria (Paulander et al. 2009), Actinobacteria (Okamoto-Hosoya et al. 2003)
Rifamycins	*rpoB*	504N	Actinobacteria (Cavusoglu et al. 2002)
		511P	Actinobacteria (Williams et al. 1998)
		512F	Proteobacteria (Barrick et al. 2010; Qiu et al. 2012; Miskinyte and Gordo 2013)
		513L	Actinobacteria (Taniguchi et al. 1996)
		516G	Proteobacteria (Barrick et al. 2010), Actinobacteria (Williams et al. 1998)
		516Y	Proteobacteria (Barrick et al. 2010), Actinobacteria (Mokrousov et al. 2003)
		526Y	Proteobacteria (Miskinyte and Gordo 2013), Actinobacteria (Taniguchi et al. 1996; Paluch-Oles et al. 2009), Firmicutes (Mariam et al. 2004)
		526L	Actinobacteria (Williams et al. 1998)
		526P	Actinobacteria (Williams et al. 1998)
		529L	Actinobacteria (Makadia et al. 2012)
		529H	Proteobacteria (Miskinyte and Gordo 2013), Firmicutes (Watanabe et al. 2011)
		531F	Proteobacteria (Miskinyte and Gordo 2013), Actinobacteria (Taniguchi et al. 1996)
		564L	Proteobacteria (Qiu et al. 2012)
		572F	Proteobacteria (Miskinyte and Gordo 2013), Actinobacteria (Siu et al. 2011)
Quinolones	*gyrA/parC*	83/80F	Proteobacteria (Kehrenberg et al. 2009), Bacteroidetes (Oh et al. 2001), Actinobacteria (Eaves et al. 2002), Firmicutes (Ricci et al. 2004)
		83/80L	Proteobacteria (Silva et al. 2011), Actinobacteria (Eaves et al. 2002; Park et al. 2011; Silva et al. 2011), Bacteroidetes (Oh et al. 2001), Firmicutes (Janoir et al. 1996)
		83/80Y	Proteobacteria (Kehrenberg et al. 2009), Actinobacteria (Eaves et al. 2002), Firmicutes (Kaneko et al. 2000)
		87/84N	Proteobacteria (Saenz et al. 2003), Actinobacteria (Eaves et al. 2002), Firmicutes (de la Campa et al. 2004)
		87/84Y	Proteobacteria (Saenz et al. 2003; Kehrenberg et al. 2009; Silva et al. 2011), Bacteroidetes (Oh et al. 2001), Actinobacteria (Eaves et al. 2002), Firmicutes (de la Campa et al. 2004)
		87/84G	Proteobacteria (Saenz et al. 2003; Kehrenberg et al. 2009; Silva et al. 2011), Bacteroidetes (Oh et al. 2001), Actinobacteria (Eaves et al. 2002), Firmicutes (Janoir et al. 1996)

The housekeeping genes targeted by the three studied antibiotic classes are highly conserved among bacteria of different phyla. Their RDRs are also relatively highly conserved (supplementary fig. S1, Supplementary Material online). Even within paralogs GyrA and ParC, when aligned, the RDRs are highly conserved (Ishiguro et al. 2006). Known TRAs to each of the three antibiotic classes are summarized in table 1. As can be seen, many of these TRAs are known to confer resistance across highly diverse bacterial phyla.

In many metagenomic studies, large assemblages of bacteria are sequenced directly from their natural environments. In such studies, high-throughput sequencing techniques are used to sequence relatively short snippets of the genomes of the bacteria residing within an environment. These short reads then often undergo an assembly process in which overlapping segments are used to produce longer stretches of contiguous sequences more ideal for assignment of phylogeny and other bioinformatics analyses.

Here, we extracted large numbers of RpsL, RpoB, and GyrA/ParC protein sequences from metagenomic data collected from a large variety of environments. Environments could be classified as host-associated (meaning samples were extracted from within the bodies of animal or human hosts) versus non host-associated. They could further be classified based on expected levels of antibiotic exposure (table 2). Once we extracted the sequences of the housekeeping genes from each environment, we examined these sequences for the presence of resistance alleles at known positions. We found that TRAs to all three antibiotics segregate at notable frequencies. TRA frequency is highest to quinolones and lowest to rifamycins. The frequency of different TRAs varies greatly, and frequencies of TRAs also vary as a function of the phylogenetic classification of the sequences and of the environment from which they were extracted. Quinolone TRA frequency is particularly alarmingly high in host-associated environments, where, on average, approximately 40% of bacteria were found to carry a quinolone TRA. Interestingly, we find high frequencies of quinolone TRAs even within hosts that were never clinically exposed to this antibiotic. At the same time, we find only relatively low frequencies of quinolone TRAs within non host-associated environments, irrespective of expected levels of antibiotic contamination.

**Table 2:** Summary of Metagenomic Datasets Analyzed in This Study

Environment	Environment Type^a^	Expected Antibiotic Exposure	No. Samples	No. of Gyra/ParC Sequences	No. of RpsL Sequences	No. of RpoB Sequences
Waste	non host-associated	High	5	256	45	150
Aquatic	non host-associated	Low	58	3,299	1,567	1,488
Soil	non host-associated	Low	27	1,092	503	529
Human gut	host-associated	High	84	1,273	1,895	611
Human tongue	host-associated	High	73	2,124	624	848
Human vagina	host-associated	High	5	114	98	82
Dog gut	host-associated	High	2	106	107	90
Insects	host-associated	Low	23	756	861	824
Hoatzin Cecum	host-associated	Low	6	161	66	120

^a^Host-associated samples were extracted from within the bodies of human or animal hosts.

## Materials and Methods

### Data Sources

The host-associated and non host-associated metagenomic datasets used in these analyses were downloaded from the Integrated Microbial Genomes with Microbiome Samples database (IMG/M) (Markowitz et al. 2012). A summary of these data is given in table 2 and supplementary table S1, Supplementary Material online. The Vallés et al. (2014) data that contain 87 unassembled gut metagenomes of infants at five stages of development and their mothers before and after birth were also downloaded from IMG/M.

### Identifying RpsL, RpoB, and GyrA/ParC Sequences

*Escherichia coli* K12 RpsL (GenBank:BAE77949.1), RpoB (GenBank:YP_491474.1), and GyrA (GenBank:NP_416734.1) protein sequences were used in the BLAST searches of the metagenome samples. The inclusion criteria of a 60% identity and empirically determined bit scores (170 for GyrA*,* 160 for RpsL, and 170 for RpoB) for BLAST searches were sufficiently low so as to not bias the housekeeping sequence selection toward Proteobacteria. In the case of RpoB, the full sequence was not used due to its extensive length (1,342 residues) relative to the average read length of the metagenome assembled reads (∼200 residues). Using the full RpoB sequence produced many results without the RDR. Instead, a 200 amino acid length sequence from position 420 to 620 that encompassed the RDR was used. In the case of GyrA and ParC, it was impossible to differentiate between the two sequences within the region of interest between the two based on BLAST results due to the short read lengths of most metagenomic sequences. However, because both have alignable RDRs with identical resistance mutations in a diverse array of bacteria (table 1, Ishiguro et al. 2006; Park et al. 2011), they were put in the same group.

### Identifying TRAs

Each metagenomic sequence was pairwise aligned to the *E. coli* reference sequence used in the initial BLAST search using the pairwise2 module included in Biopython (http://biopython.org/DIST/docs/api/Bio.pairwise2-module.html). The BLOSUM-62 scoring matrix was used with identical open and extend gap penalties for each alignment. Because of strong homology, misalignments did not occur (which was verified by manual inspection of the alignments). The query sequence was used as a reference in the alignment to identify the column position of each possible TRA within the pairwise alignment. Results were maintained in a pandas DataFrame of all the sequences and their amino acids at the resistance-conferring sites.

### Phylogenetic Profiling

Phylogenetic profiling was conducted on each group of GyrA/ParC, RpsL, and RpoB sequences collected from each sample. First, for each of the housekeeping genes, we constructed a BLAST database composed of all the complete bacterial protein sequences of that gene within the NCBI RefSeq database. BLASTp was then used to compare the sequences of GyrA/ParC, RpsL and RpoB found within metagenomic samples to their respective RefSeq BLAST databases. Only hits with an *E* value of e-20 or lower and an identity of at least 60% were recorded. Based on these BLAST results, the phylum level classification of each metagenomic sequence was assigned according to the phylum level classification of the RefSeq sequence to which it was found to be most similar.

Phylum level assignment of all sequences within each of the host-associated and nonhost-associated samples described in table 2 was carried out using the built-in IMG/M program. A threshold of at least 60% identity of all sequences with their annotated matches was imposed (Markowitz et al. 2008). Phylogenetic profiling of the unassembled mother–infant pairs was taken from Vallés et al. (2014).

### Testing the rpoB and gyrA Genes for Signals of Recombination

The Phi test that is implemented within the Phipack package (Bruen et al. 2006) was used to examine whether the *rpoB* and *gyrA* genes showed significant signals of recombination within each of the tested bacterial species. A *P* value of 0.05 was required to reject the null hypothesis that a gene within a bacterial lineage was clonal using a window size of 100 and 1,000 permutations. If a gene lacked enough informative sites, no classification was made. A minimum multiple sequence alignment length of 900 bp was required, and thus *rpsL* was not included in this test.

### Statistical Analyses

All statistical analyses were carried out using R (R Core Team 2014).

Log linear analysis was carried out using the “glm” function (see supplementary text S1, Supplementary Material online). Mann–Whitney tests were carried out using the wilcox.test function. FDR *P*-value corrections were carried out using the p.adjust function.

## Results

### Notable Frequencies of Antibiotic Resistance Alleles within Most Examined Environments

The protein sequences of the *E. coli* K12 RpsL, RpoB, and GyrA/ParC were compared against 283 metagenomic datasets from the IMG/M database, belonging to nine different environment types (table 2, supplementary table S1, Supplementary Material online). Sequences that matched RpsL, RpoB, or GyrA/ParC beyond a certain threshold (see Materials and Methods) were further considered. Environment types studied can be divided into host-associated and non host-associated. Host-associated samples are defined as those extracted from within human or animal bodies. The environment types can further be divided into those that are expected to be more or less contaminated by antibiotics. Host-associated environments expected to be more exposed to antibiotics include samples extracted from humans and dogs. Host-associated environments expected to be less exposed to antibiotics include samples extracted from the tropical pheasant Hoatzin (as these samples were extracted from the wild [Godoy-Vitorino et al. 2008] and insect samples). Non host-associated environments expected to be more exposed to antibiotics are represented by samples extracted from waste treatment facilities. At the same time, samples extracted from soil or aquatic environs are likely to be contaminated with antibiotics to a lesser extent. In table 2, we summarize the nine studied environment types, the number of samples of each type, the number of sequences found for each of the housekeeping genes in each environment, and the classification of the environment types according to host-association and expected level of antibiotic exposure. It is important to note that we cannot be sure and do not rely on an assumption that any of the environments studied is devoid of antibiotic contamination.

Quinolones target two paralagous proteins, GyrA and ParC. The sequences of these two proteins are virtually identical across their first 300 amino acids. It is therefore not possible to reliably distinguish these two genes from metagenomic short reads. However, the same alleles have been shown to confer resistance in both GyrA and ParC (table 1). We therefore combined all sequences that align to GyrA, referring to them as GyrA/ParC, and calculated the frequency of resistance within both these genes combined.

The metagenomic sequences identified as GyrA/ParC, RpoB, or RpsL were checked for antibiotic resistance alleles previously shown to confer resistance (table 1). We remind the reader that we refer to such resistance alleles occurring within the housekeeping target genes of the antibiotics as TRAs. All studied TRAs are located in regions of the housekeeping genes that are highly conserved across bacterial phyla (supplementary fig. S1, Supplementary Material online). For many of the examined TRAs, it has been demonstrated that they confer resistance across diverse bacterial phyla (table 1).

**Fig. 1.—:**
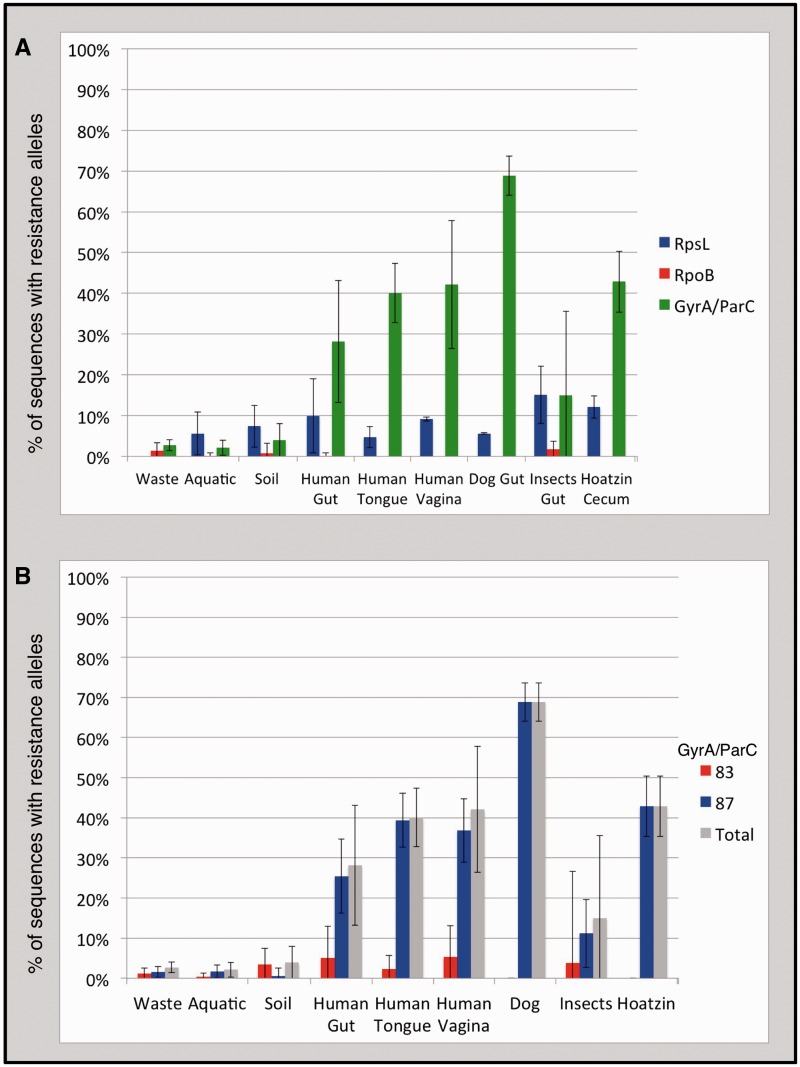
Average segregation frequencies of TRAs to three antibiotic classes among bacteria residing within nine sampled environment types. (TRAs are defined as resistance alleles occurring within the housekeeping targets of antibiotics.) For each environment type, the average resistance allele frequency across all samples is depicted. Error bars represent standard deviations. (*A*) Frequency of TRAs to three broad-spectrum antibiotic classes: quinolones (green, TRAs found in the *gyrA* or *parC* genes), rifamycins (red, TRAs found in the *rpoB* gene), and streptomycin (blue, TRAs found in the *rpsL* gene). (*B*) Frequency of quinolone TRAs broken down by position within the GyrA/ParC protein sequences. Proportion of sequences carrying a quinolone TRA at any position is given in gray. Proportion of sequences carrying a quinolone TRA at position 83 of GyrA or 80 of ParC is given in red. Proportion of sequences carrying a quinolone TRA at position 87 of GyrA or 84 of ParC is given in blue.

**Fig. 2.—:**
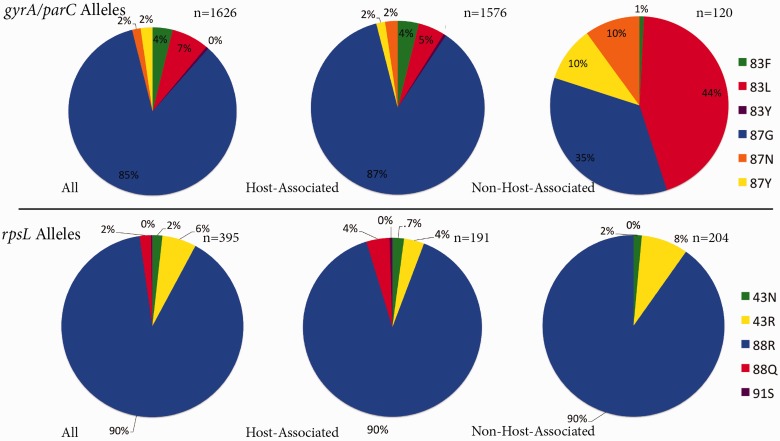
Breakdown of quinolone and streptomycin TRAs among sequences carrying a TRA. Depicted are three pie charts for each antibiotic class, representing the distribution of TRAs within all environments, host-associated environments, and non host-associated environments. *n* denotes the total number of sequences with TRAs in each group.

The first striking result of these analyses is the observation of a notable frequency of TRAs to all three antibiotics across most environments (fig. 1*A*). Rifamycin TRAs are segregated at the lowest frequencies. The frequency of RpoB sequences carrying a rifamycin TRA is on average 0.49% across environments and ranges between 0% and 1.70% for the different environments analyzed (fig. 1*A*). RpsL sequences carry resistance alleles to streptomycin with an average frequency of 7.44% across studied environments, ranging between 0% and 15.10% (fig. 1*A*). Across all environments sampled, resistance to streptomycin was conferred almost exclusively by a single resistance allele, arginine at RpsL position 88 (88R, position numbers are given in reference to the *E. coli* amino acid sequence, fig. 2). Quinolone TRAs segregate at the highest frequencies, with an average frequency across environments of 17.78%, ranging between 2.12% and 68.87% (fig. 1*A*).

### Extremely High Frequency of Quinolone TRAs Particularly within Hosts

Within host-associated environments, an extremely high proportion of GyrA/ParC sequences carried quinolone TRAs (fig. 1*A*). This proportion ranged from 15% to 68.87% in the six different host-associated environments examined, with an average of 39.5%. These frequencies are much higher than those observed in non host-associated environments, where the frequency of quinolone TRAs ranged between 2.15% and 3.93%. These results are quite striking, as they seem to indicate that the frequency of quinolone TRAs is higher within hosts, irrespective of expected levels of antibiotic exposure. To further examine this, a Log Linear Model was constructed which attempted to explain frequencies of quinolone TRAs based on expected antibiotic exposure and host association. This model showed that both expected antibiotic exposure and host association significantly affect the frequency with which TRAs are found within an environment (for more details on the model, see supplementary text S1, Supplementary Material online).

While the results of the model indicate that both antibiotic exposure and host association affect quinolone TRA frequencies, it appears that the effect of host association may be more pronounced. Frequencies of quinolones resistance TRAs were significantly higher in all host-associated environments compared with all non host-associated environments (FDR-corrected *P* < 0.05 for all comparisons, according to an unpaired one-tailed Mann–Whitney test, supplementary table S2, Supplementary Material online). At the same time, when examining host-associated environments, it is not always the case that those expected to be less exposed to antibiotics contain quinolone TRAs less frequently. The frequency of quinolone TRAs within the tropical pheasant Hoatizn microbiome (that was collected in the wild [Godoy-Vitorino et al. 2008]) was not significantly lower than that observed within the human gut, tongue, or vagina (*P* > 0.05 for all comparisons, supplementary table S2, Supplementary Material online). Similarly, when examining non host-associated environments, only 2.73% of GyrA/ParC sequences found within waste samples (expected to be relatively highly contaminated with antibiotics) carried quinolone TRAs. The relatively low frequencies of quinolone TRAs found within waste samples are not significantly higher than those found in soil and are only marginally significantly higher than those found in the aquatic samples (FDR-corrected *P* = 0.673 and FDR-corrected *P* = 0.05, respectively, supplementary table S2, Supplementary Material online). As already mentioned, the frequency of quinolone TRAs within waste samples is significantly lower than those found even within those host-associated environments that are expected to be less exposed to antibiotics (i.e., insects [FDR-corrected *P* = 0.02] and Hoatzin [FDR-corrected *P* = 0.003]).

In addition to frequencies of quinolone TRAs, patterns of quinolone TRAs also vary between host-associated and non host-associated environments. Resistance to quinolones within hosts is conferred almost exclusively (∼87% of the time) by a specific resistance allele, glycine at position 87 of GyrA or 84 of ParC (87/84G, position numbers are given in reference to the *E. coli* amino acid sequence) (figs. 1*B* and 2). At the same time, in non host-associated environments, the 87/84 G allele is less dominant and is found in only approximately 35% of sequences carrying a quinolone TRA (figs. 1*B* and 2).

Combined, these results demonstrate that there is an association between expected levels of antibiotic exposure and frequencies of quinolone TRAs. However, host association also exerts a highly significant effect on quinolone TRA frequency. Indeed, irrespective of expected levels of antibiotic exposure, within hosts, there is a much higher frequency of quinolone TRAs compared with what is observed within non host-associated environments. Furthermore, within but not outside of hosts, resistance is conferred almost exclusively by a single TRA, GyrA/ParC position 87/84 G.

### TRA Frequencies and Patterns Vary Greatly between Phyla

We phylogenetically classified the identified sequences of RpsL, RpoB, and GyrA/ParC. To perform this classification, each sequence was assigned a phyla level classification based on its closest match in NCBI’s Reference Sequence database (RefSeq) using BLASTp. Because of the relatively short length of the metagenomic sequences and the high levels of conservation of the examined housekeeping gene sequences, only phyla level classification was feasible. To insure that the phylogenetic proportions of housekeeping gene sequences extracted from each sample were relatively representative of the sample as a whole, phylogenetic profiles were generated based on all sequences found within each environment (Materials and Methods). The phylogenetic profiles obtained based on each housekeeping gene could then be compared with those obtained using the entire datasets. There was some expected variation in certain profiles (supplementary fig. S2, Supplementary Material online), especially between environments with low numbers of RpsL, RpoB, and GyrA/ParC sequences, but overall, the phylogenetic profiles generated based on the three housekeeping genes were congruent with those generated based on all sequences within their environments.

**Fig. 3.—:**
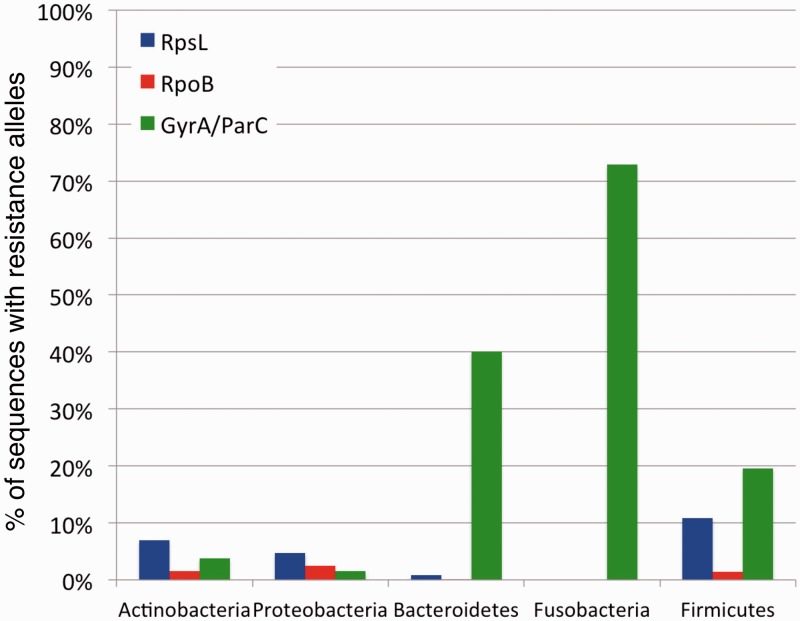
Frequency of TRAs varies greatly between different bacterial phyla. Depicted are the average frequencies with which GyrA/ParC (green), RpoB (red), and RpsL (blue) protein sequences carry resistance alleles for the five most prevalent phyla. These frequencies are calculated based on combined data from all environments sampled.

Following phylogenetic classifications of sequences of the three housekeeping genes, we could examine the frequency of TRAs within each phyla separately (fig. 3). We find that different phyla vary in overall frequencies of TRAs (fig. 3). While also present for the other antibiotic classes, this variation in TRA frequency is most noticeable in the case of quinolone resistance. GyrA/ParC sequences classified as belonging to Fusobacteria, Bacteroidetes, and Firmicutes carry quinolone TRAs much more frequently than sequences classified as coming from Proteobacteria or Actinobacteria (fig. 3).

**Fig. 4.—:**
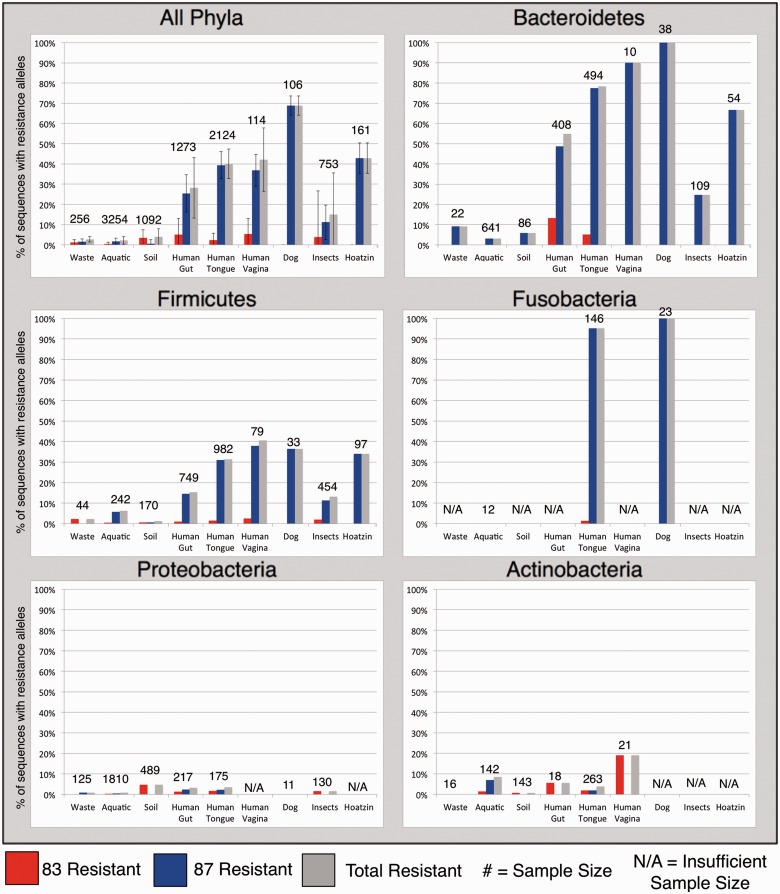
Frequency quinolone-resistance alleles partitioned by phylogeny and environment. Depicted are the average frequencies with which *gyrA/parC* genes carry a quinolone resistance allele (gray), the frequency with which they carry a resistance allele at position 83/80 (red), and the frequency with which they carry a resistance allele at position 87/84 (blue). Note that in almost all cases in which a position 87/84 resistance allele is present, the resistance allele is 87/84G (fig. 2). For resistance allele frequency to be displayed for a given phylum in a given environment, at least ten GyrA/ParC sequences had to be found for that phylum in that environment. The marking N/A is used to mark cases in which less than ten sequences were found.

Next, for each of the five most frequent phyla, we examined how quinolone TRA frequency varied across the different environments examined (fig. 4). For these analyses, we only considered a given phylum in a given environment if we could find at least ten GyrA/ParC sequences belonging to that phylum in that environment. We found that for the three phyla that carry quinolone TRAs most frequently (Fusobacteria, Bacteroidetes, and Firmicutes), quinolone TRA frequency tended to be much higher within hosts. Resistance within these phyla is conferred almost exclusively by the 87/84G allele (fig. 4). In contrast for the two phyla that were less frequently found to carry TRAs (Actinobacteria and Proteobacteria), the difference between host-associated and non host-associated environments was far less clear (fig. 4).

Combined, our results show that different phyla vary in their propensity to carry TRAs to quinolones (and to a lesser extent also to streptomycin and rifamycins). When combined with the results of previous sections, these results suggest that higher frequencies of quinolone resistance via the 87/84G allele found within hosts are driven by the increased tendency of Bacteroidetes, Firmicutes, and Fusobacteria to acquire and/or maintain this TRA within hosts. Additionally, these elevated TRA frequencies are also driven by the fact that host-associated environments are enriched for these three phyla (supplementary fig. S2, Supplementary Material online).

### High Frequencies of Quinolone TRAs within Human Samples Not Clinically Exposed to Antibiotics

**Fig. 5.—:**
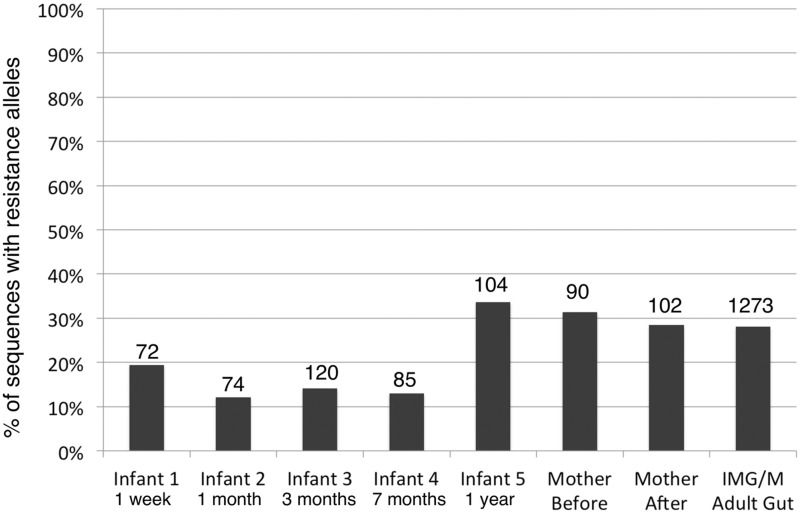
Frequency of GyrA/ParC sequences harboring Quinolone TRAs within the Vallés et al. (2014) cohort of mother/infant gut metagenomes.

We already showed that quinolone TRA frequencies were quite high, even in hosts that were not expected to be clinically exposed to antibiotics (i.e., the tropical pheasant Hoatzin, for which ∼43% of GyrA/ParC sequences carried a TRA). To further demonstrate that frequencies of TRAs can be quite high within hosts—even in the absence of clinical antibiotic exposure—we focused on gut samples from humans we know were not clinically treated recently or at all with quinolones and the remaining antibiotic classes on which we focused in this study. Vallés et al. sequenced gut metagenomic samples from mother–infant pairs. The mothers were sampled before and after giving birth, and the infants were sampled along five time points of development during the first year following their birth. Antibiotic usage was documented for both the mothers and the infants. For this reason, we know that the mothers were not treated with our antibiotics of interest for at least 3 months prior to giving birth and that the children were never directly treated with these antibiotics. Although, these metagenomic samples were not directly exposed to antibiotics in clinical dosages, we found a relatively high frequency of quinolone TRAs within both mothers and children (ranging from 12.16% to 33.65% with an average of 22.56%, fig. 5). Quinolone TRA frequencies within the mothers and the oldest children match very well the frequency of TRAs we observed in the human gut samples extracted from IMG/M (fig. 5).

As in our previous results, we found that different phyla showed distinct frequencies of quinolone TRAs (supplementary fig. S3, Supplementary Material online). As before, higher frequencies of TRAs were found in Bacteroidetes and Firmicutes, compared with lower frequencies in Actinobacteria and Proteobacteria. An outlier of this general trend was found in week-old infants where we observed a relatively high frequency of quinolone TRAs within Proteobacteria (19.23%). Also, as before, resistance in Bacteroidetes and Firmicutes was conferred predominantly by the GyrA/ParC 87/84G allele.

Vallés et al*.* (2014) observed a distinct and dramatic phylogenetic shift in the studied infants, from a Proteobacteria-heavy microbiome postbirth to a microbiome of primarily Bacteroidetes and Firmicutes, more reminiscent of that of the adult mothers, after 1 year. We also observed this shift in our collected housekeeping gene sequences. As different phyla differ greatly in their propensity for carrying TRAs, these shifts in phylogenetic composition were also accompanied by changes in the frequency of quinolone TRAs (fig. 5). As can be expected from the fact that Bacteroidetes and Firmicutes are more frequent in mothers and older children and from the fact that these phyla more often carry quinolone TRAs within hosts, we observe higher frequencies of quinolone TRAs in the oldest children and in the mothers. As mentioned above, these TRA frequencies match very well what we observed in the IMG/M human gut samples (fig. 5).

Less expectedly, we find that the frequency of streptomycin TRAs also increases in the oldest children; 13.59% of all bacteria extracted from 1-year-old infants carried a resistance allele within RpsL (fig. 5). The increase in streptomycin TRA frequency with age was most notable for Proteobacteria, where a small majority of bacteria were seen to harbor streptomycin TRAs in the oldest, 1-year-old cohort of infants (52.38%, supplementary fig. S3, Supplementary Material online). Such high frequency of streptomycin TRAs is not found within the mothers, before or after birth, or within IMG/M adult gut microbiomes. Frequencies of rifamycin TRAs remain relatively low and unremarkable within both infants and mothers, as is the case with assembled IMG/M gut results.

Unlike the datasets summarized in table 2, the sequences from the mother–infant gut samples of the Vallés et al. study were unassembled. The fact that the results obtained from both type of data were largely consistent demonstrates that biases introduced in the assembly process have not greatly affected the trends we observed.

Together, these results demonstrate that TRAs segregate at high frequencies even within the gut microbiomes of humans that were never exposed to clinical antibiotic usage. TRA frequency is particularly high in the case of quinolones, to which the predominant TRA within the human gut is the 87/84G allele. Also, consistent with the results presented in previous sections, the 87/84G allele is found much more often within Bacteroidetes and Firmicutes and less frequently within Actinobacteria and Proteobacteria.

### GyrA/ParC Sequences Carrying Two Separate TRAs Can Be Found Almost Exclusively within the Human Gut

GyrA sequences carrying TRAs at both the 83 and 87 position are known to confer compounded resistance to quinolones relative to bacteria with a single resistance allele at either position (Conrad et al. 1996; Oh et al. 2001). When examining the frequency of IMG/M-extracted GyrA/ParC sequences carrying resistance alleles at both positions, we found that they occur predominantly within the human gut, where they appear at a frequency of 2.34%. Such sequences were most frequent in Bacteroidetes (6.25%).

Next, we examined the frequency of GyrA/ParC sequences carrying double TRAs within the Vallés et al. mother–offspring gut dataset. We found that in the mothers, the frequency of sequences carrying double TRAs were comparable to those found in the IMG/M gut datasets. Five of the mothers’ GyrA/ParC sequences carried two resistance alleles (2.49%). These five sequences were all classified as Bacteroidetes, meaning that 6.94% of Bacteroidetes GyrA/ParC sequences found within the mothers’ guts carried two TRAs. In contrast, we found no GyrA/ParC sequences carrying double TRAs within the guts of the infants.

## Discussion

This study demonstrates that resistance alleles occurring within the housekeeping targets of antibiotics (which we refer to as TRAs) segregate at alarmingly high frequencies within natural bacterial populations. Such high frequencies of segregation of TRAs may increase the likelihood of pathogenic bacteria obtaining such alleles via recombination with resistant, nonpathogenic bacteria sharing their environments. The risk of dispersal of resistance between nonpathogenic and pathogenic bacteria varies between horizontally transferred resistance genes but is clearly quite high for some (reviewed in Martinez et al. [2015]). When it comes to TRAs, it is unclear whether housekeeping target genes of antibiotics undergo sufficient recombination to make the possibility of recombination a real threat. To examine this, we extracted from the NCBI, the protein coding gene sequences and annotations of members of bacterial species for which there were at least ten sequenced strains. From these data, we extracted the sequences of the *gyrA* and *rpoB* genes and used the Phipack software package (Bruen et al. 2006) to test whether within each species there is any signal that these genes have recombined. (We could not carry out a similar analysis for the *rpsL* gene due to its short length.) We excluded from our analysis four species for which our analyses of recombination and/or prior knowledge show that there is in general no or very little recombination within the species. These species were *Yersinia pestis*, the *Mycobacterium tuberculosis* cluster (MTBC), *Francisella tularensis,* and *Corynebacterium pseudotuberculosis.* This left us with seven remaining species that included *E. coli*, *Clostridium botulinum, Bacillus cereus, Streptococcus suis, Streptococcus pneumonia, Corynebacterium diphtheria*,** and *Neisseria meningitides.* We found that GyrA showed a significant signal of recombination within three of these species: *E. coli* (*P* ≪ 0.001), *C. dip*h*theria* (*P* ≪ 0.001), and *B. cereus* (*P* = 0.002). Fitting with these results, a previous study has also demonstrated that the *gyrA* and *parC* genes have undergone recombination within clinical isolates of Streptococcus pneumoniae (Ferrandiz et al. 2000). RpoB only showed signals of recombination within one of the species, *N. meningitides* (*P* ≪ 0.001). These results demonstrate that even when looking at a relatively small collection of bacterial species, there are signs that the housekeeping genes targeted by antibiotics can indeed recombine. Hence, it is possible for pathogenic bacteria to acquire TRAs from nonpathogenic bacteria with which they cohabitate. High frequency of quinolone TRAs within host microbiomes may therefore pose a threat for the acquisition of resistance by pathogenic bacteria.

The frequency of TRAs varied greatly between antibiotic classes. Frequencies of TRAs were highest for quinolones and lowest for rifamycin. This is in sharp contrast to the numbers of TRAs examined, with the potential to confer resistance to each antibiotic class, which were highest for rifamycins and lowest for quinolones (table 1). Furthermore, this is also in contrast to the rates with which resistance develops in laboratory evolution experiments in *E. coli* and other closely related Proteobacteria. In such experiments, resistance develops more rapidly toward rifampicin (a rifamycin) and more slowly toward nalidixic acid (a Quinolone) and streptomycin (Bjedov et al. 2003; Paulander et al. 2009; Katz and Hershberg 2013).

A particularly high frequency of quinolone TRAs was observed in this study (17.91% on average). Quinolone TRA frequency varied greatly as a function of environment (ranging between 1.47% and 72.90%) and differed clearly between host-associated and non host-associated environments. Host-associated environments were characterized by much higher frequencies of quinolone resistance alleles. TRA frequencies in such environments were highest within certain phyla (Bacteroidetes, Firmicutes, and Fusobacteria), and resistance was conferred almost exclusively by the GyrA/ParC position 87/84 G allele. In contrast, within non host-associated environments, quinolone TRAs segregated at much lower frequencies. These trends were found to be largely independent of expected levels of clinical antibiotic exposure. For example, the patterns of segregation of TRAs described here for host-associated environments were found in gut samples extracted from a wild pheasant and in gut samples extracted from a large cohort of children that were never clinically treated with quinolones. At the same time, patterns of segregation of TRAs observed for non host-associated environments were found to hold for waste/sewage environments that are expected to face high levels of antibiotic contamination.

It is very possible (and even likely) that clinical exposure to antibiotics does affect the frequency with which TRAs segregate within bacterial populations. Yet, our results strongly suggest that such exposure is not the sole determinant of the frequency with which such resistance alleles will be found within different bacterial phyla and within different environments. Indeed, clinical antibiotic exposure may not even be the strongest determinant of TRA frequency. For one, if clinical exposure were the dominant factor driving TRA frequencies to be higher within host-associated environments, we might expect all phyla to be affected. Yet, we only observed increased abundance of TRAs within some phyla and not others. Additionally, if antibiotic exposure was the sole determinant of the observed higher segregation frequency of TRAs, we would expect all resistance alleles to be affected. Under such a model, we would expect higher frequencies of the GyrA/ParC position 87/84 N and Y quinolone resistance alleles and of the position 83/80 resistance alleles to be found within hosts as well. Yet, the higher frequency of quinolone TRAs observed within hosts is explained almost entirely by the higher frequency of a single resistance allele (87/84G). Finally, if exposure to antibiotics is the dominant factor driving higher quinolone TRA frequencies within hosts, we would expect the microbiomes of hosts that were less exposed to antibiotics to show lower frequencies of TRAs. As discussed above, this was not always the case.

It is possible that subclinical exposure to antibiotics could contribute to the increased abundance of TRAs observed within hosts. Although, it is not quite clear why a tropical pheasant would be more exposed to quinolones than waste water, and why this would lead to such different patterns of TRA segregation than observed within non host-associated environments. It is also possible that hosts that were not directly exposed to antibiotics have high frequencies of quinolone TRAs because of possible high rates of horizontal spread of strains among individuals. Such high spread could lead to the transfer of strains that carry TRAs from hosts that were exposed clinically to antibiotics to hosts that were never exposed. This would mean that the high frequencies of quinolone TRAs found within hosts that were never directly exposed to clinical doses of quinolones are still the indirect result of quinolone exposure. It is important to note, however, that this model would require extremely high rates of horizontal spread to explain how the oldest infants that have never been exposed to quinolones have frequencies of quinolone TRAs that are so similar to those observed within their mothers and other adult guts. Additionally, this model would require that tropical pheasants also horizontally acquire very large numbers of strains that carry TRAs due to quinolone exposure. It is unclear how this would occur, given that the pheasant samples were reportedly extracted from the wild (Godoy-Vitorino et al. 2008).

Another factor that seems likely to affect the frequency of TRAs in the manner found here is antibiotic-independent selection acting on these alleles. GyrA, ParC, RpoB, and RpsL are all important housekeeping proteins. Resistance alleles within these proteins change their structure and likely also their function. These changes to such important proteins are quite likely to have functional effects on the bacterium carrying them, independently of whether this bacterium is exposed to antibiotics. It is largely assumed that resistance alleles within these proteins most often tend to reduce bacterial fitness in the absence of antibiotic exposure (Andersson and Hughes 2010). For a while, it was hoped that such negative fitness effects of resistance alleles would lead to a reduction in their frequency once antibiotic exposure ceased. More recently, this optimistic view was put into question with the discovery that compensatory mutations could alleviate the deleterious effect of resistance alleles (reviewed in Andersson and Hughes [2010]). Additionally, it has been shown that some TRAs carry no fitness effect, while others can even be beneficial to the bacteria carrying them, under certain growth conditions (Wrande et al. 2008; Paulander et al. 2009; Kunz et al. 2012; Katz and Hershberg 2013; Miskinyte and Gordo 2013; Qi et al. 2014). Indeed, we were able to demonstrate that the GyrA position 87G allele (that we find here to be the most frequent within hosts) carries a growth advantage in starved *E. coli* microcolonies (Katz and Hershberg 2013).

Experiments looking at the fitness effects of resistance alleles tend to be carried only in very specific bacteria and are almost always limited to the Proteobacterium *E. coli* and its close relatives. Such experiments are usually also limited to only a few specific growth conditions. Yet, these experiments clearly demonstrate that the fitness effects of TRAs vary greatly and are greatly affected by growth conditions (Wrande et al. 2008; Paulander et al. 2009; Katz and Hershberg 2013; Miskinyte and Gordo 2013). It is quite reasonable to assume that both the fitness effects of TRAs and the capability to compensate for deleterious effects of such alleles could vary greatly between phyla and as a function of environment. Such variation in the antibiotic-independent fitness effects of TRAs could very well contribute to the great variation we observe in the frequency of these alleles within different environments and among different phyla. For instance, the high frequency of the GyrA/ParC 87/84 G allele within specific phyla in host-associated environments might indicate that this allele may confer on bacteria belonging to these phyla some sort of substantial growth advantage within hosts. Variation in the antibiotic-independent fitness effects of TRAs could also explain the differences we found in the frequency of TRAs conferring resistance toward the three different antibiotics studied. Our results therefore imply that to understand the causes behind increases in antibiotic resistance frequency, we will need to obtain a much better understanding of how resistance alleles in general and TRAs in particular affect bacterial fitness in the absence of antibiotic exposure.

It is important to note that it is possible that some of the examined TRAs do not universally confer a resistance phenotype (measurable by examining MIC) on the bacteria in which they reside. For example, it is possible that a certain TRA will only confer resistance if it is found in some bacterial species but not in others. At the same time, most examined quinolone TRAs (including the most frequently observed GyrA/ParC, 87/84G allele) have been shown to confer resistance in a diverse array of species belonging to the four phyla we found to be most abundant in our analyses (Proteobacteria, Bacteroidetes, Actinobacteria, and Firmicutes [table 1]). It therefore seems highly likely that these TRAs indeed confer a resistance phenotype in species belonging to these phyla. Even if in some cases TRAs do not confer resistance, if a sequence carrying a resistance allele were to recombine into a pathogenic species in which such an allele does confer resistance it could lead to resistance in that pathogen. Therefore, high frequencies of segregation of TRAs within hosts could have clinical repercussions even if these resistance alleles do not universally lead to a resistance phenotype.

Not all TRAs examined here can confer full resistance to clinical dosages of the antibiotics they protect against. This is not to say that they cannot be clinically relevant and increase the likelihood that a pathogen carrying these alleles will survive antibiotic treatment and develop compound resistance. For example, the accumulation of bacteria carrying two different TRAs, conferring clinical levels of resistance may occur much more easily on the background of such high frequencies of single TRAs. Supporting this concern, we observed that within the human gut, Bacteroidetes have a rather high frequency of ParC/GyrA sequences carrying two separate TRAs (6.25%).

To summarize, our results show an alarmingly high frequency of quinolone TRAs within the bodies of humans and other animals. In addition, we find notable, albeit lower, frequencies of streptomycin and rifamycin TRAs. A specific quinolone resistance allele (GyrA/ParC position 87/84 G) segregates at very high frequencies within Firmicutes, Bacteroidetes, and Fusobacteria within hosts. This resistance allele has been previously shown to confer resistance to quinolones across highly diverged phyla, including Bacteroidetes and Firmicutes (table 1). Finally, our results show that higher frequencies of quinolone TRAs within hosts do not seem to be the result of direct clinical antibiotic exposure.

## Supplementary Material

Supplementary text S1, tables S1 and S2, and figures S1–S3 are available at *Genome Biology and Evolution* online (http://www.gbe.oxfordjournals.org/).
